# From a Surgeon Dentist's Case Book

**Published:** 1852-10

**Authors:** J. L. Levison

**Affiliations:** Brighton, England.


### ARTICLE IV.

*From a Surgeon Dentist's Case Book.* By J. L. Levison, D. D. S., Brighton, England.

Messrs. Editors:—Not having time just now to give you an Essay I had proposed sending for your excellent journal, and wishing to give proof of my fealty and adhesion to you, for the eminent services you have done to render the practice of dentistry what it ought to be—like a grateful creditor, as it is not in my power to pay you what it was my intention to do, I will throw myself on your recognized courtesy, and ask you for the present to accept a few miscellaneous cases as an instalment. These cases are taken from a large number in my note book, and although they will not have any actual connection, they may still possess some little interest, whilst they will have *one* great merit—brevity; and will, therefore, not take a long time to read.

Abscess in the Fang.—A gentleman of a nervo-sanguinous temperament, called on me in a state of intense excitement, to extract for him the second molar on the right side of the lower jaw. He had stopped it himself with either Thomas' or Howard's wonderful, most wonderful, cement or succedaneum, which is sold in bottles with ample directions to make "every man his own dentist."*

The poor fellow's agony and impatience were commensurate. He stamped his feet—swore he should go mad! His face was flushed, particularly on the side of the affected tooth—his eyes were blood-shot, and his expression haggard and jaded.

The tooth was tender to the touch, and quite black, whilst the external surface or anterior portion of the alveolus was distended, like as if there had been a deep seated abscess. After some little time, as the tooth was very brittle, I extracted it.

* Most likely the sage and philosophic personages were plagiarists in this instance, as there is a work, "every man his own Farrier."

One fang was highly vascular, and on removing the mercurialized tin, (amalgam,) the internal and lower extremity of the affected fang had a considerable quantity of *pus* within it, if the contracted space is taken into consideration.

In this case it may be presumed, that my patient suffered from a two-fold cause, viz. the presence of the crystallized amalgam, from its hardness and roughness irritating the surface of the nerve—vascular pulp on which it rested; and, secondly, from the evidence of galvanic action, having converted the free oxygen in the saliva into an acid, as the crown and fangs had scarcely a trace of lime; and at the same time that it had induced a succession of shocks, and thus by reflex action affected the brain, and spinal system.

Comment is unnecessary. I would simply remark, that such cases have long satisfied me that my views on the injurious effects of amalgams and alloys, (published in the Lancet, Sept. 11, 1831,) were founded on sound scientific and physiological data.

Paralysed eye-lids from dental irritation.—On Thursday, May 22nd, 1851, I met in the course of my morning's walk, Miss S——, the principal of a boarding school for young ladies, leading one of her pupils, as if she were totally blind. As the young lady in question was a patient of mine, for some dental deformity, I naturally expressed sorrow for her, and some surprise at the suddenness of the attack. On opening the eye-lids forcibly, and shading her eyes from the light, (as we were on the street,) I observed that they did not appear bright but dull, and besides there was a want of polish about them. In reply to my question "can you see clearly?" she answered, "no, all things appear to be dim, as if a mist was over them." It was then stated by Miss S—— that her pupil had been so for five days, and that she had become very anxious about her—proof of which she gave of an almost maternal solicitude, by the gentle manner she led her from place to place. The young lady was about thirteen years old, of a nervous temperament, average size brain, very fair complexion, light grey eyes, and almost flaxen hair. But there was a great torpor and feebleness in her constitution. This latter circumstance was obvious by the tardiness which marked her second dentition.

I strongly recommended that an eminent oculist should see her, but a preference was given for their own medical attendant. They went to him immediately and he prescribed for her, and they called on me afterwards on their way home—as the younger one was under my professional care.

The incisors and cuspidati had some of them presented their lateral sides, and hence gave an unpleasant expression, particularly as they were so irregular that some stood out and some within the alveolar arch, making a sort of zig-zag at the anterior portion of the mouth. On the present occasion I took an opportunity of inspecting the whole mouth, when I observed the second molar of the right side, in the upper jaw, still deeply seated within the socket, the gum over it and the adjoining parts, including a portion of the palate and cheek, presenting a highly vascular condition, the blood-vessels being literally gorged. She did not, however, complain of any particular pain on pressing these parts, but having had much experience of similar cases,* it occurred to me, that in all probability the motar nerves of the eye-lids were affected from sympathy, arising from buccal disturbance, (another instance of reflex nervous action,) and that they were not the result of any local affection to account for their suspended function, as the eye-lids themselves were in a normal condition. Hence, I proposed lancing the gum, which appeared indurated, and in consequence the confined tooth was not only held "in durance vile," other organs were implicated. Scarcely had I finished making the crucial incision, by carrying the edge of the gum-lancet sufficiently deep so as to come in direct contact with the superior surface of the crown itself, when instantaneously the young lady was disenchanted; and her spell-bound eyes were once more under her volition. She opened them, stared, smiled, and then expressed her joy that she now could use them without either effort or annoyance.

*I have since my residence in Brighton had some cases from the Eye Infirmary.

It may be added, that the paralysis of the lids did not occur again, though she had misgivings from time to time. But every few days I repeated my scarification to prevent the wounds from cicatrizing, which might have again induced the irritation and a relapse.

I also took the precaution, as the eye-lashes were almost colorless, to paint them with a black pomade, and the result was so beneficial, that her vision was stronger, from the better endurance of the light.

Indurated parotid gland.—A gentleman consulted me, the latter end of the last year, "as he suffered continued pain in his mouth." He was a tall, thin man, with a good brain and active temperament, but a feeble digestion, and of literary habits; and his general health was affected in consequence of these combined causes, which were greatly aggravated by his sedentary occupation. All the gums were much inflamed and spongy—the teeth generally were loose. And the dens sapientiæ on the right side deeply imbedded in the gum, as there had not been space sufficient for its development. It was carious and rough, and acted as an irritant to the whole inner portion of the cheek on the same side. This led me to notice the enlarged and indurated parotid gland, which latter had caused him much anxiety. I removed the affected tooth, and attended to his mouth, which I cured. And there is every reason to anticipate that the affected and swollen gland will disperse, as the last time I saw my patient it was already softer, smaller, and less conspicuous.

Osseous union of the two temporary central incisors. The coachman of my friend Dr. Pickford, of this town, brought one of his children to have some teeth extracted. The mouth had a curious appearance. The two central incisors were close together, being literally "two in one," and on examining the alveolar process, the septum had either been absorbed, or else it had never existed. Neither of the fangs had the slightest absorption, so that they had effectually presented a powerful barrier to their successors, who were forced sideways, and seemed in the first instance to be the lateral and the central ones, as they subsequently proved to be. After removing the teeth, the space was very great, and I merely made this note of it, from reflecting, that in all probability the two rudimentary *follicles* had been primarily in contact, and they had proceeded to their perfect development, as if there had been but one *rudimentary* follicle.

OSSIFIED GUMS.—A very nice and respectable middle aged woman, the nurse in a gentleman's family, called to have a molar tooth removed, the last in her jaw on the right side. On endeavoring, by the ordinary method, to separate the gum from the neck of the tooth, there was a most unusual resistance—the gum being as hard as bone itself, whilst the color was also paler than usual, having scarcely the slightest tinge of red. In fact the gum had, instead of its normal structure, been changed into a something of a pseudo-osseous substance, neither bone nor cartilage. By means of a flat instrument and the application of some force, it was loosened from the neck of the tooth, but not before it had been slightly slit in two places; at which places *spicula* were observed, and thus its relation to a true bony structure was rendered obvious. The tooth was afterwards removed with comparative ease, but there followed a copious hemorrhage, which, however, was soon subdued. She called a few days after the operation, and which enabled me to examine the substance more leisurely. She had not any pain, but complained that "the gums had a peculiar dead feel," and on the same side they were quite colorless.

I record the fact as curious, because in a rather extensive practice in twenty-seven years, I have never seen an exactly similar case. And it is a matter of certainty, that without the precaution of having separated the ossified gum before attempting to extract the tooth, that the probability is that the substance itself would have been frightfully lacerated.

